# Rotator Cuff Tendon Dimensional Variability, Novel Patient-Specific Measurement Method—Morphological Measurement for Rotator Cuff Tendon

**DOI:** 10.3390/jcm12237307

**Published:** 2023-11-25

**Authors:** Raphael Lotan, Mojahed Sakhnini, Ariel Oran, Oded Hershkovich

**Affiliations:** 1 Department of Orthopedic Surgery, Wolfson Medical Center, Sackler School of Medicine, Tel Aviv 5265601, Israel; dr.lotan@gmail.com; 2 Department of Orthopedic Surgery, Sheba Medical Center, Sackler School of Medicine, Tel Aviv 5262000, Israel; mojahed.sakh@gmail.com (M.S.);

**Keywords:** rotator cuff, humeral head, measurement, relative, size

## Abstract

Introduction: The shoulder rotator cuff (RC) is crucial to shoulder function and involvement in shoulder pathology. RC tears have been extensively studied, and several classifications have been devised to quantify their magnitude. Various RC measurement techniques were introduced previously, utilizing cadaveric specimens, X-rays, CT scans, and MRI with different results published regarding humeral heads’ different plane diameters and the correlation to age, gender, and height. There are very few studies measuring RC length in the general population. Purpose: We aimed to assess the geometrical relation between rotator cuff tendon length and humeral head sagittal and axial diameters. Methods: A total of 100 shoulder MRI scans of labral tear-suspected patients were reviewed, and the geometrical parameters of the rotator cuff length and proximal humerus sagittal and axial diameters were measured. Results: The healthy population has wide variability in humeral diameter and rotator cuff length. We found a high correlation between humeral head sagittal and axial plane diameters and the rotator cuff tendon dimension. The orthogonal plane diameters disagree with the humeral head being round but rather spheric. The rotator cuff length changes according to the patient’s gender and height. Conclusion: This is a novel method for rotator cuff measurement, description, and classification according to the percentage of tear instead of length (cm). This method is more clinically oriented and relevant than most other previous methods.

## 1. Introduction

The shoulder rotator cuff (RC) is crucial to shoulder function and involvement in shoulder pathology [1]. Rotator cuff tears have been extensively studied, and several classifications have been devised to quantify their magnitude (small, medium, or large). A less than 2 cm RC tear is considered a small tear, 2–4 cm a medium tear, and >4 cm a massive tear [2]. Only a few studies considered the size difference between males and females or the correlation between patient height, humeral head size, and rotator cuff dimension [3]. Previous cadaveric anatomic studies included old-aged individuals [4,5], but degenerative changes might have affected humeral head geometry; thus, biasing measurements, also differences in measurement techniques can also affect results. Height and weight are also biased as they can change with age. Iannotti et al. studied cadaver shoulders and patient MRIs, the cadaveric cohort’s mean age was 75 years, and the patient’s average age was 38 years; both cohorts had the same average height of 175 cm [5]. The humeral head radius of curvature was similar, precluding age as a significant factor in humeral diameter [6]. There was, however, wide variability in humeral head size with a direct correlation to height in men and women. Several humeral head diameter measurement methods were described [4,5,7,8], including direct measurements on cadaveric specimens [9], patients’ radiographs [10], digitized images taken in the scapular plane [11], computerized tomographic data and three-dimensional computer modelling [12], and MRI [5]. Boileau et al. found less than a 1 mm difference in coronal and axial plane curvature in 88.2% of the 65 humeral cadaveric specimens [4]. Iannotti et al. demonstrated a two-millimetre difference, on average, between axial and coronal plane curvatures in both cadavers and patients [5]. Cadaveric specimen measurements were performed manually using a calliper. Hertel et al. showed a mean 12% difference between the coronal and sagittal humeral head curvatures, but measurements were performed on standardized X-rays, which are still inherently inaccurate and could undermine his results [9]. Iannotti et al. investigated proximal humeral geometry on 96 cadaveric humeri measurements and 44 patient MRIs. Both groups showed a high linear correlation between both radii regarding the humeral geometry and linear regression analysis of R = 0.985 [5]. This result confirms that MRI is comparable to direct cadaveric humeral measurements and the negligible effect of age. However accurate these measurements are relevant for humeral head arthroplasty pre-operative planning [7,12], they may still be irrelevant for evaluating the rotator cuff tendon width concerning the humeral head. The third table in Section 3 shows the results of the different studies.

Dugas et al. investigated the area and dimensions of the rotator cuff tendons and their distance from the articular surface on 20 fresh-frozen cadaveric upper extremity specimens. They did not find a correlation between the humeral head and rotator cuff insertion dimensions [12]. The RC dimensions in the general population are poorly described, reducing the sensitivity of the current RC tear classification systems.

The study hypothesis is that rotator cuff size is patient specific and related to sex, height, and humeral head dimension, and this study aims to evaluate the RC dimension’s correlation to sex, height, and humeral head size determined via MRI.

## 2. Materials and Methods

The study cohort included one hundred proximal humeri of patients undergoing MRIs (1.5 Tesla GE HDxt MRI Scanner, Amsterdam, The Netherlands) for clinical suspicion of a labral tear, as found using positive O’Brien active compression test, biceps load test, and clunk and crank tests. Exclusion criteria included a history of humeral head fractures, avascular humeral head necrosis, infection, or malignancy. Cases with humeral head defects, precluding measurements, such as a Hill–Sachs lesion, were also excluded. Radiographic measurements were performed using the PACS software (Picture Archiving and Communication System; Version: 5.11.0-0150). The universal PACS image storage and transfer format is DICOM (Digital Imaging and Communications in Medicine). For every case, gender, age, weight, height, and MRI findings were collected. MRI studies were evaluated independently by a board-certified radiologist.

**Figure 1:**
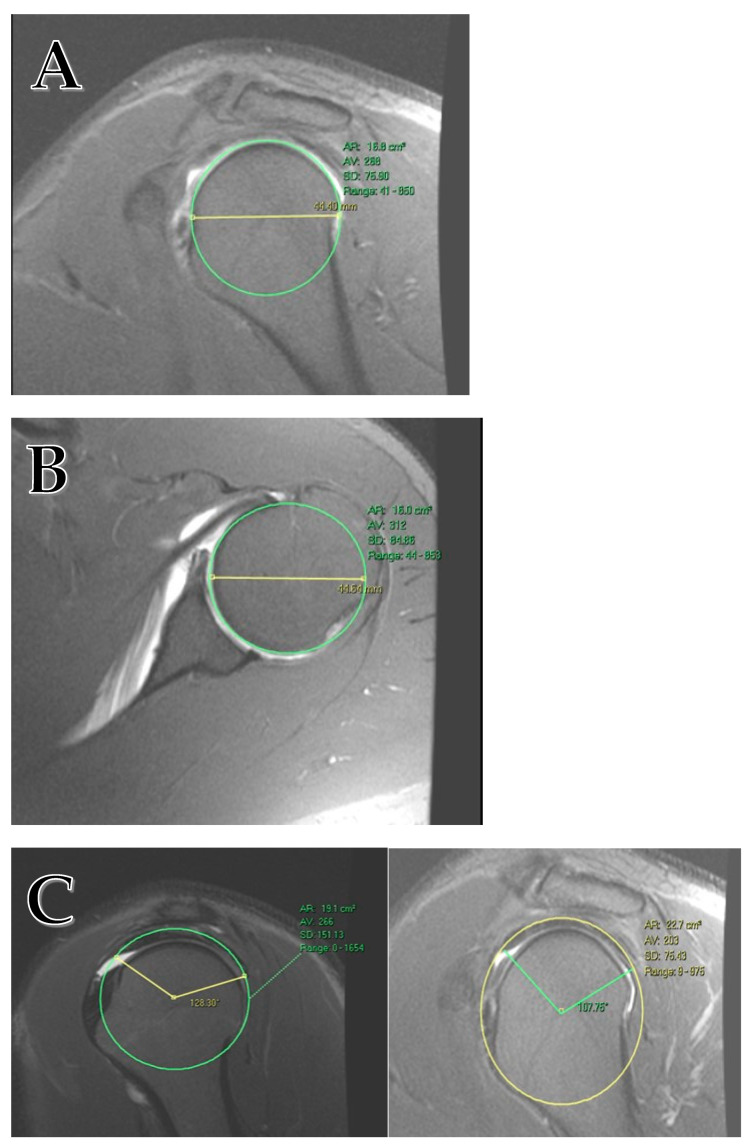
(**A**) Humeral head diameter measurement on the sagittal plane. (**B**) Humeral head diameter measurement on the axial plane. (**C**) The rotator cuff tendon is measured on the sagittal plane on the image of the largest head diameter. The RC length is calculated using trigonometry. The tendon has a hypointense signal that reflects the confluence of the supraspinatus and infraspinatus tendon.

Anatomical measurements included maximal sagittal humeral head diameter (HumSG; Figure 1A), axial humeral head diameter (HumAX; Figure 1B), and the supraspinatus and infraspinatus tendon length from anterior to posterior (AP_SS_IS; Figure 1C). Rotator cuff tendon length was measured on the sagittal plane using an image depicting the greatest head diameter; the length was calculated using trigonometry. The RC measured is the confluent tendon of the supraspinatus and infraspinatus as they merge to insert into their footprint. All measurements were taken using T2 weighted MRI images (T2WI). Measures were re-evaluated and reconfirmed by a second author.

Exclusion criteria included RC tears, humeral head fractures, or deformities, such as avascular necrosis, osteomyelitis, and malignancies involving the humeral head. Hill–Sachs lesions were not excluded, and measurements were conducted using the best fit of a circle to the unaffected humeral head.

Our institutional review board approved this study.

### Statistical Analysis

Statistical analysis was performed using IBM SPSS 23.0 software.

Descriptive statistics were used to assess the study population. Results are presented as a percent for categorical variables and mean ± standard deviation for continuous variables. The Student’s *t*-test was used to compare variables between males and females. Pearson correlation analysis was used to delineate the relation between humeral head parameters and the relation between the rotator cuff length and the different variables.

Linear regression was used to find the relation between the sagittal and axial diameters of the humeral head. Multi-variate regression analysis was conducted between the rotator cuff tendon as a dependent variable and the other independent variables.

A statistically significant difference was considered when the *p*-value was equal to or less than 0.05.

## 3. Results

The study cohort included 100 participants, 89 males and 11 females, with similar ages between the male and female groups, 25.4 and 26, respectively, *p* = 0.77. Gender height and weight differences were as expected, 177.3 and 164.6 cm, respectively (*p* < 0.001), and 77.1 and 57.6 kg, respectively (*p* < 0.001). BMI followed the same gender differences, *p* < 0.001 (Table 1).

The cohort’s MRI findings included 49% SLAP lesions, 23% Hill–Sachs lesions, 19% anterior labral tears, 17% Bankart lesions, and 6% acromioclavicular joint pathologies.

Humeral head size differed among males and females; the mean sagittal humeral head diameter was 45.8 mm in males and 40.8 mm in females, *p* < 0.001, and the axial diameter was 47.5 mm and 40.5 mm, respectively, *p* < 0.001 (Table 1). The male sagittal humeral head diameter was 5 mm longer in males than in females and 7 mm longer axially.

The average anterior–posterior supraspinatus–infraspinatus length was 59.9 mm in males and 53.5 mm in females, *p* < 0.001 (Table 1). The rotator cuff tendon length was 6.4 mm longer in males than in females.

**Figure 2:**
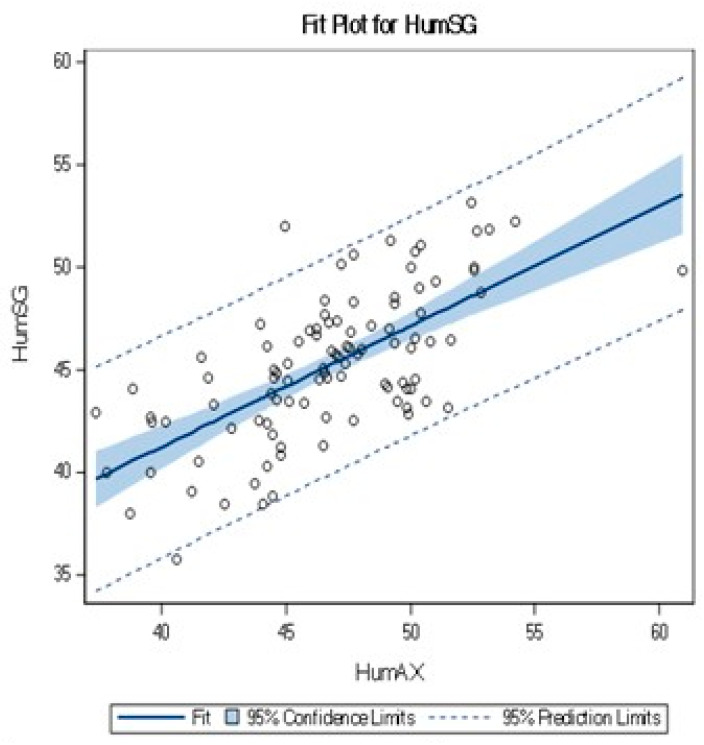
Linear correlation between HumSG and HumAX.

Figure 2 demonstrates measurements dispersal between the sagittal and axial humeral head diameters. A correlation was found between the humeral head sagittal and axial diameters, R = 0.66. The relation between the measured diameters is presented in the following equation: HumSG = 17.73 + 0.58 × HumAX. The symmetrical dispersal of measurements on either side of the trend line is consistent with the linear trend.

**Figure 3:**
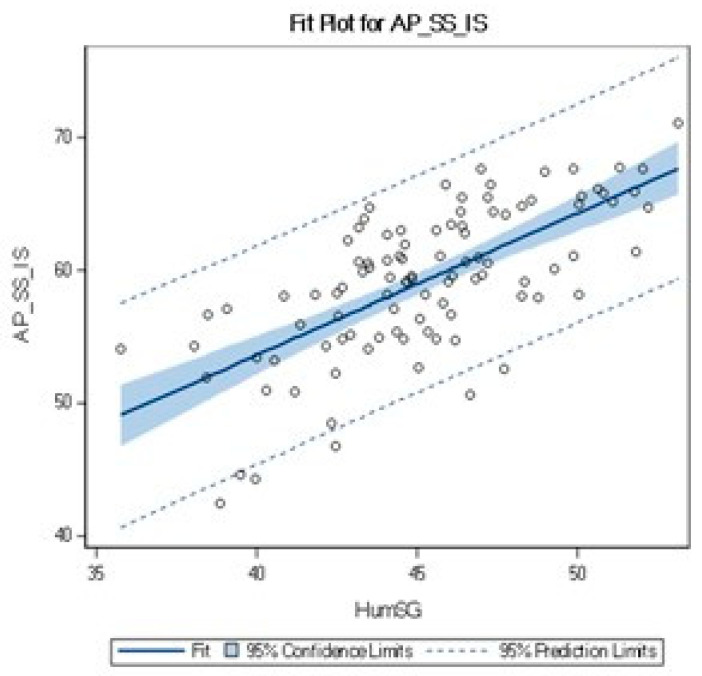
Linear relation between the AP_SS_IS and HumSG.

Another correlation was found between the sagittal humeral head and the rotator cuff tendon dimension (R = 0.68) (Table 2). Figure 3 shows that measurements are symmetrically dispersed around the linear trend line.

Height and weight were moderately or mildly related to the RC tendon dimension (Table 3). Multi-variate linear regression analysis with the RC length as the dependent variable and sagittal humeral diameter, age, height, weight, and gender as independent variables demonstrate that only HumSG is correlated with AP_SS_IS, *p* < 0.0001 (Table 3).

## 4. Discussion

A better understanding of rotator cuff size variability amongst the healthy population may improve patient-specific evaluation of RC pathologies. Correlating the RC length to a static, reproducibly measurable anatomic structure, such as the humeral head, is a means to provide an objective patient-specific definition of the precise anatomical RC length and the actual percentage of RC tear.

This study demonstrates that height, weight, BMI, humeral head sagittal, and axial diameters are significantly different between males and females (Table 1), as previously described [4,5,7], yet still debated [12]. As expected, age was not found to be related to the humeral head diameter or RC length.

We found a high correlation between sagittal and axial plane humeral head diameters, R = 0.66. The mean sagittal plane diameter was 45.2 mm (35.8–53.1 mm), and the axial plane diameter was 46.8 (37.4–60.9 mm). The average sagittal and axial plane paired difference was 1.5 mm. These results demonstrate the correlation between the two orthogonal plane humeral head diameters but disclaim the spherical nature of the humeral head.

Different from Dugas et al. [12], which did not find a correlation between the humeral head and rotator cuff insertion dimensions, this study found a correlation between the sagittal humeral head and rotator cuff tendon dimension, R = 0.68 (Table 2). These findings add up anatomically according to the population’s humeral head diameter variability.

In our study, the largest humeral head diameter was 60.9 mm, and the smallest was 37.4 mm, making a 23.5 mm or a 61% difference. The RC tendon length ranged from 71 mm to 42.5 mm, a 28.5 mm difference. Thus, a 2 cm RC tear in a 4.3 cm RC is a considerable tear involving 46% of tendon length, while the same tear in a 71 mm RC involves only 26% of tendon length. Although not as easy, the relative way of describing the tear as a percentage of the involved RC tendon length is more accurate in describing the severity of the tear than mere centimetres classification. These results bear significance in approaching rotator cuff tears and proximal humerus arthroplasty among males and females.

This study’s limitations lie in the low percentage of females included, the two-dimensional approximation needed with the technique described, and the inclusion of Hill–Sachs lesions into this study that may affect the humeral head measurement. Using the best-fit circle to the humeral head allows a good enough approximation of humeral head diameter regardless of Hill–Sachs lesions. Care must be taken not to include [9] the teres minor and subscapularis in the measurements. Teres minor insertion on the greater tuberosity is not on the same sagittal plane image as the supraspinatus.

The diameter of curvature in our study in axial and sagittal plane concord with prior studies [4,5,8,9,10,11,12] (Table 4).

## 5. Conclusions

In conclusion, we describe the general population’s variability in humeral head sagittal and coronal diameters. Humeral head diameters are affected by the patient’s gender and height. The same variability and correlation exist in rotator cuff tendon length. We introduce a novel method of classifying patient-specific rotator cuff dimensions based on humeral head geometry measured on MRI imaging that can be further utilized to classify and manage rotator cuff tears; the absolute size of an RC tear should be evaluated in light of the described patient-specific RC size when contemplating treatment.

We hope this method can contribute to radiological standardization of MRI rotator cuff tear measurement and further elaborate the indication for surgical repair of rotator cuff tears.

## Figures and Tables

**Table 1:** Patient characteristics.

	Male (*n* = 89)	Female (*n* = 11)	*p*-Value	All (*n* = 100)
Age (years)	25.4 ± 6.4	26 ± 7.6	0.80	25.4 ± 6.5
Height (cm)	177.3 ± 6.3	164.6 ± 41.4	<0.001	175.9 ± 7.4
Weight (kg)	77.1 ± 14.3	57.5 ± 4.5	<0.001	75 ± 14.9
BMI	24.5 ± 3.9	21.3 ± 1.9	<0.001	24.1 ± 3.8
HumSG (mm)	45.8 ± 3.2	40.8 ± 2.9	<0.001	45.2 ± 3.5
HumAX (mm)	47.5 ± 3.4	40.5 ± 2.5	<0.001	46.8 ± 4
AP_SS_IS (mm)	59.9 ± 5.1	53.5 ± 5.8	0.004	59.2 ± 5.6

HumSG = maximal sagittal humeral head diameter, HumAX = axial humeral head diameter, AP_SS_IS = supraspinatus and infraspinatus tendon length from anterior to posterior.

**Table 2:** Pearson correlation coefficients.

Pearson Correlation Coefficients, *n* = 100						
	BMI	Weight	Height	Age	AP_SS_IS	HumSG
AP_SS_IS	0.199	0.365	0.493	0.038	1.000	0.681
*p*-value	0.0475	0.0002	<000.1	0.709		<000.1

**Table 3:** Pearson correlation coefficients by BMI, weight, height, and age.

Pearson Correlation Coefficients, *n* = 100						
BMI	Weight	Height	Age	AP_SS_IS	HumSG	
0.19873	0.36516	0.49347	0.03776	1.00000	0.68164	AP_SS_IS
0.0475	0.0002	<000.1	0.7091		<000.1	*p*-value

**Table 4:** Humeral head diameter in previous studies.

Study	Specimen	Humeral Head Diameter (mm)			Humeri Examined (*n*)
		Min	Mean	Max	
Boileau et al. [4]	cadaveric	37.1	46.2	56.9	65
Iannotti et al. [5]	cadaveric	36	44	54	96
	patients	38	46	56	44
Dugas et al. [12]	cadaveric	34	46	56	60
Hertel et al. [9]	cadaveric	34	42	56	200
Irlenbusch et al. [10]	radiographs	48.6	54.08	59.56	106
Sharkey et al. [11]	cadaveric	46.1	50.6	57.5	5
Zuckerman et al. [8]	cadaveric	49.6	?	51.1	29

Zuckerman mentioned only the median (50.3).

## Data Availability

The complete data are available under a confidentiality restriction.
